# TEST: A Z, A&Z, A<Z, A°Z, A Z, A±Z, AµZ End test. Analysis of soft tissue injuries associated with distal radius fractures

**DOI:** 10.1186/2052-1847-5-19

**Published:** 2013-09-02

**Authors:** Takeshi Ogawa, Toshikazu Tanaka, Takaji Yanai, Hiroshi Kumagai, Naoyuki Ochiai

**Affiliations:** 1 Department of Orthopaedic Surgery, Kikkoman General Hospital, 100 Miyazaki, Noda, Chiba 278-0005, Japan

## Abstract

**Background:**

Soft tissue injuries associated with distal radius fractures have been reported by some authors. Arthroscopy can be used to evaluate the condition of the articular surface and intracarpal soft tissues and as an aid to treatment. There are three intracarpal soft tissues of particular importance: the triangular fibrocartilage complex (TFCC), the scapholunate interosseous ligament (SLIL), and the lunotriquetral interosseous ligament (LTIL). The purpose of this study was to evaluate intracarpal soft tissue injuries and their relationships with fracture types during arthroscopic surgical treatment of distal radius fractures.

**Methods:**

Eighty-nine patients in our hospital underwent surgical treatment by arthroscopy for a fracture of the distal end of the radius. They ranged in age from 17 to 92 years (mean, 62.2 years), and comprised 20 men (mean age, 45.6 years) and 69 women (mean age, 66.5 years). The AO classification data on plain radiographs indicated A2 fracture in four patients, A3 fracture in 10 patients, C1 fracture in two patients, C2 fracture in 24 patients, and C3 fracture in 49 patients. Wrist arthroscopy was performed with vertical traction, and intracarpal soft tissues were examined. For assessment of the TFCC in the radiocarpal space, the Palmar classification was used. For assessment of the SLIL and LTIL in the midcarpal space, the Geissler classification was used.

**Results:**

TFCC injury was present in 59% of cases, SLIL injury in 54.5% of cases, and LTIL injury in 34.5% of cases. Only 17.1% of patients (14/82 patients) were negative for all three types of injury. In 81% of cases (72/89 patients), some intracarpal soft tissue injury was present in association with the fracture.

**Conclusions:**

The fracture was complicated by TFCC injury in 59% of patients, SLIL injury in 54.5% of patients, and LTIL injury in 34.5% of patients, irrespective of the fracture type.

## Background

The factors determining the treatment outcomes of distal radius fractures include shortening, angle of reduction, articular congruence, and soft tissue injuries. There are three intracarpal soft tissues of particular importance: the triangular fibrocartilage complex (TFCC), the scapholunate interosseous ligament (SLIL), and the lunotriquetral interosseous ligament (LTIL). These tissues cannot be fully detected on standard radiographs of wrists with distal radius fractures [1]. Geissler et al. [2] evaluated 60 cases with intra-articular fractures using arthroscopy, and reported that TFCC injury was present in 43% of cases, SLIL injury in 31% of cases, and LTIL injury in 15% of cases. They classified the SLIL and LTIL injuries into Grades I–IV. However, Rimington et al. [3] evaluated fresh-frozen cadavers with normal wrists, and recognized many cases with grade II or III instability. These ligament laxities may have been derived from inherent ligament laxities.

We combine arthroscopy with all surgical procedures for distal radius fractures, and use it to assist in the evaluation of soft tissue injuries and the treatment of such fractures. Although the focus of the study was not on the actual treatment of these fractures, it is important to provide a brief description of the technique used for the treatment. In most patients, the fracture was treated with manipulative reduction and plate fixation or external fixation. The purpose of this study was to summarize the frequencies of the soft tissue injuries associated with distal radius fractures, and to investigate the relationship between extra-articular fractures and intra-articular fractures.

## Methods

Institutional review board approval was obtained by the ethics committee of our hospital, and patients provided written informed consent to participate in the study.

Between December 2008 and July 2012, 89 patients in our hospital underwent surgical treatment by arthroscopy for a fracture of the distal end of the radius. They ranged in age from 17 to 92 years (mean, 62.2 years), and comprised 20 men (mean age, 45.6 years) and 69 women (mean age, 66.5 years). The fractures involved 30 right wrists and 59 left wrists. The AO classification data on plain radiographs indicated A2 fracture in four patients, A3 fracture in 10 patients, C1 fracture in two patients, C2 fracture in 24 patients, and C3 fracture in 49 patients. The types of fracture were dorsal flexion dislocation in 71 patients, volar flexion dislocation in 15 patients, and intermediate type in three patients.

The operations were performed by the first author (T.O.) or second author (T.T.) using a modified Geissler method [2]. The wrist was suspended in a traction tower, and 10 pounds of traction was applied through finger traps to the thumb and middle or ring finger. A 23-gauge needle was inserted between the extensor pollicis longus tendon and extensor digitorum communis tendons (3/4 portal), and the joint was distended using saline. The skin was then lanced over the 3/4 portal by pulling the skin against a number 15 scalpel blade, and a hemostat was used to dissect down to the joint capsule to prevent injury to the cutaneous nerves and dorsal veins. A blunt arthroscopic cannula was inserted through the portal, and the joint was lavaged to clear it of hematoma and fracture debris. A small-joint arthroscope (1.9 mm wide) was inserted through the cannula in the 3/4 portal. An additional portal was created between the extensor digitorum communis tendons and extensor digiti minimi tendon (4/5 portal). A small-joint shaver (2.9 mm wide) placed through this portal was used to help to clear the joint of any remaining hematoma and provide a good view of the fracture site. Loose fragments of bone and cartilage were removed with a mini-grasper. Extravasation of fluid through the fracture fragments and capsular rents that could cause troublesome soft tissue swelling were minimized by allowing the irrigation fluid to exit through the arthroscopic cannula or a separate outflow portal. The midcarpal space was also evaluated by placement of the arthroscope approximately 1 cm distal to the 3/4 portal, in line with the radial border of the long finger in the radial midcarpal space. The inflow cannula was initially left in the radiocarpal space after the arthroscope had been placed in the midcarpal space to determine whether the irrigation fluid communicated with the midcarpal space, which would suggest a possible tear of an interosseous ligament. Subsequently, the inflow was transferred to the arthroscopic cannula, and a needle for outflow of the irrigation fluid was placed in the ulnar midcarpal portal located 1 cm distal to the 4/5 portal, in line with the metacarpal of the ring finger. Injuries to the interosseous ligaments between the carpal bones of the proximal carpal row were determined by observing separation of the bones from each other and/or incongruity of their distal articular surfaces.

For assessment of the TFCC in the radiocarpal space, the Palmer classification was used [4]. In this classification, lesions thought to result from a traumatic injury are considered to be class 1. The class 1 lesions are subdivided into those with a central perforation (type A), a peripheral tear (type B), a tear distal to the articular disk (type C), or a tear of the triangular fibrocartilage from the sigmoid notch of the radius (type D). Class 2 lesions, which are degenerative injuries, are thought to result from a traumatic injury if perforation of the disc (C–E) is detected. Clinically, there may not be major differences between class 2A–2B lesions and class 2C–2E lesions. We considered that Class 2C–2E lesions were often accompanied by synovial hyperplasia around the TFCC disc tear, which required debridement.

For assessment of the SLIL and LTIL in the midcarpal space, the Geissler classification was used [2]. In this classification, lesions thought to result from a traumatic injury are considered to be unstable Grades II–IV. Grade II lesions were left untreated, while Grade III–IV lesions were treated by transcutaneous pinning.

A Kruskal–Wallis test was used to evaluate the relationships between the fracture types and soft tissue injury types. Values of *P* < 0.05 were considered to indicate statistical significance.

## Results

TFCC injury was present in 59% of cases (49/83 patients), SLIL injury in 54.5% of cases (48/88 patients), and LTIL injury in 34.5% of cases (30/87 patients) (exclusive of non-assessable patients). Only 17.1% of patients (14/82 patients) were negative for all three types of injury. In 81% of cases (72/89 patients), some intracarpal soft tissue injury was present in association with the fracture. The frequencies of the fracture types and intracarpal soft tissue injuries are summarized in Table 1. Although the population was small and scattered, TFCC, SLIL, and LTIL injuries were detected irrespective of the AO or intracarpal/extracarpal classifications of the fractures (Table 1). The treatments for TFCC according to the Palmer classification were as follows [5]: 1a (27 cases) and 2 (15 cases), debridement only; 1B with distal ulnar fracture (1 case), tension band wiring; 1B without distal ulnar fracture (1 case), 1C (0 cases), and 1D (5 cases), above elbow splint fixation. The treatments for SLIL and LTIL according to the Geissler classification were as follows [6]: I–II (39 and 25 cases), no treatment; III (6 and 5 cases), percutaneous pinning; IV (3 and 0 cases), dorsal ligament reconstruction.

**Table 1:** Intracarpal soft tissue injury frequencies according to the fracture types

**AO classification**	**TFCC injury**	**SLIL injury**	**LTIL injury**
A2	50% (2/4)	75% (3/4)	50% (2/4)
A3	80% (8/10)	50% (5/10)	30% (3/10)
C1	50% (1/2)	50% (1/2)	0% (0/2)
C2	52% (11/21)	67% (16/24)	38% (9/24)
C3	59% (27/46)	48% (23/48)	34% (16/47)
total	59% (49/83)	54.5% (48/88)	34.5% (30/87)
p-value	0.47	0.55	0.79

## Discussion

In this study, we did not aim to advocate the use of arthroscopy for treatment of distal radius fractures in the wrist. Instead, we found that arthroscopy was a valuable and sensitive adjunct for determining the prevalence and severity of intracarpal soft tissue lesions that were not detectable on standard radiographs of wrists with such fractures.

The prevalence of ligamentous injury associated with displaced fractures of the distal part of the radius has been shown to be as high as 98%, with 27 of 50 fractures (54%) in one study being associated with a tear of the scapholunate (SL) ligament [7]. Richards et al. [8] reported that the TFCC was torn in 46 of 118 patients, and occurred in 35% of intra-articular fractures and 53% of extra-articular fractures. No correlation between ulnar styloid fractures and TFCC injuries was found. SLIL injuries with instability were present in 21.5% of intra-articular fractures and 6.7% of extra-articular fractures. LTIL injuries with instability were present in 6.7% of intra-articular fractures and 13.3% of extra-articular fractures. No significant difference between extra-articular and intra-articular fractures existed with respect to the frequencies of ligamentous injury [8].

It should be considered whether the intracarpal lesions in the present study were definitely acute or whether they might have been degenerative and thus present before the distal radius fracture. In a study of 62 wrists from cadavers of individuals with an average age of 78 years (range, 46 to 99 years) at the time of death, Wright et al. [9] found SLIL lesions in 18 wrists (12 lesions were incomplete), LTIL tears in 20 wrists (8 lesions were incomplete), and TFCC tears in 33 wrists (11 lesions were central and 21 were vertical radial). Mikic [10] noted perforations of the TFCC in 53% (26/49) of his study patients, who were aged above 60 years. In addition, the arthroscopic appearances of acute and chronic tears of the TFCC differ, in that an acute central or radial tear is seen as a linear dorsal volar tear, with sharply defined edges, as opposed to a more chronic lesion, which characteristically appears as a circular defect with ill-defined and frayed edges. The presenting finding of acute peripheral tears is hemorrhagic synovitis along the ulnar periphery of the particular disk or an osseous avulsion from the sigmoid notch of the radius [4]. We considered that not all of the injuries required treatment. In TFCC injuries, Class 1B–1D injuries according to the Palmer classification may require treatment, while Class 1A or Class 2 injuries do not necessarily need treatment [5]. Furthermore, SLIL and LTIL injuries of Geissler classification Grade III and above, through which a 2 mm probe can be passed, require some treatment, while Grade II or lower injuries do not necessarily need treatment [6]. In total, only 7.2% of TFCC injuries, 10.2% of SLIL injuries, and 4.6% of LTIL injuries required treatment in our study.

In the present study, SLIL and LTIL injuries were present not only in severely comminuted intra-articular fractures, but also in minimally displaced extra-articular fractures. The pathomechanics of combined intercarpal ligamentous injuries and fractures of the radius have been proposed by Mudgal and Hastings [11]. Two mechanisms exist. One consists of compression loading with shear stress across the SLIL, and the mechanism involved in this SLIL injury is associated with die-punch fractures. The other consists of a combination of tensile stress and compression stress, resulting in a four-part fracture associated with SL injury. Although Mudgal and Hastings [11] only considered intra-articular fractures, similar mechanisms would explain the occurrence of ligamentous injuries in extra-articular fractures. Mudgal and Jones [12] described 10 fractures of the distal end of the radius associated with SL diastasis that were detected on plain radiographs. They postulated that, as the lunate is driven proximally, a shear stress is imposed on the SLIL, resulting in either attenuation or rupture. Forward et al. [13] reported that Grade III SLIL injuries can be associated with ulnar positive variance at the time of initial presentation of a distal radial fracture and increased SL joint pain after 1 year. These injuries could lead to SL dissociation at the time of follow-up, particularly in patients with intra-articular fractures [13].

The present study has some limitations. First, the numbers of subjects varied greatly among the different types of fractures, and traumatic and degenerative injuries could not be strictly differentiated from one another. Second, we did not use distal radioulnar joint (DRUJ) arthroscopy, and did not perform DRUJ arthrography. If DRUJ arthroscopy or arthrography is used, we think the incidence of TFCC injuries would be higher. Nonetheless, the present data, which were obtained with a certain set of criteria, could be useful in future operations and treatment plans. The frequency of intracarpal soft tissue injuries requiring additional treatment besides treatment for the distal radius fracture was only 10%, which was not very high. However, it does also indicate that the outcomes of fracture treatment can be unfavorable in 1 of 10 cases. To prevent these 10% of cases being overlooked, wrist arthroscopy is useful, and this technique should be mastered by hand surgeons.

## Conclusions

Intracarpal soft tissue injuries associated with distal radius fractures were evaluated by arthroscopy. The fracture was complicated by TFCC injury in 59% of patients, SLIL injury in 54.5% of patients, and LTIL injury in 34.5% of patients, irrespective of the fracture type.

## Institutional review board

Institutional Review Board of Kikkoman general hospital.

## Competing interests

The authors declare that they have no competing interests.

## Authors’ contributions

All authors read and approved the final manuscript.

## Pre-publication history

The pre-publication history for this paper can be accessed here:

http://www.biomedcentral.com/2052-1847/5/19/prepub
